# A photodetector based on the non-centrosymmetric 2D pseudo-binary chalcogenide MnIn_2_Se_4_†

**DOI:** 10.1039/d4tc04380d

**Published:** 2025-01-15

**Authors:** Marco Serra, Nikolas Antonatos, Luc Lajaunie, Josep Albero, Hermenegildo Garcia, Mouyi Weng, Lorenzo Bastonero, Kalyan Jyoti Sarkar, Rui Gusmão, Jan Luxa, Rafał Bartoszewicz, Jakub Ziembicki, Iva Plutnarová, Nicola Marzari, Robert Kudrawiec, Zdenek Sofer

**Affiliations:** a Department of Inorganic Chemistry, University of Chemistry and Technology Prague Technická 5 166 28 Prague 6 Czech Republic marco.serra@unimore.it zdenek.sofer@vscht.cz; b Department of Semiconductor Materials Engineering, Faculty of Fundamental Problems of Technology, Wrocław University of Science and Technology Wybrzeże Wyspiańskiego 27 50-370 Wrocław Poland; c Departamento de Ciencia de los Materiales e Ingeniería Metalúrgica y Química Inorgánica, Facultad de Ciencias, Universidad de Cádiz Campus Río San Pedro S/N Puerto Real 11510 Cádiz Spain; d Instituto Universitario de Investigación de Microscopía Electrónica y Materiales (IMEYMAT), Facultad de Ciencias, Universidad de Cádiz, Campus Río San Pedro S/N Puerto Real 11510 Cádiz Spain; e Instituto de Universitario Tecnología Química, Universitat Politècnica de València-Consejo Superior de Investigaciones Científicas (UPV-CSIC), Universitat Politècnica de València Avda. de los Naranjos s/n 46022 Valencia Spain; f Theory and Simulation of Materials (THEOS), École Polytechnique Fédérale de Lausanne (EPFL), Lausanne CH-1015 Lausanne Switzerland; g U Bremen Excellence Chair, Bremen Center for Computational Materials Science, and MAPEX Center for Materials and Processes, University of Bremen D-28359 Bremen Germany; h Laboratory for Materials Simulations, Paul Scherrer Institut (PSI) 5232 Villigen Switzerland

## Abstract

Due to their attractive band gap properties and van der Waals structure, 2D binary chalcogenide materials have been widely investigated in the last decade, finding applications in several fields such as catalysis, spintronics, and optoelectronics. Ternary 2D chalcogenide materials are a subject of growing interest in materials science due to their superior chemical tunability which endows tailored properties to the devices prepared thereof. In the family of A^II^B^III^_2_X^VI^_4_, ordered ZnIn_2_S_4_-like based photocatalytic systems have been studied meticulously. In contrast, reports on disordered phases appear to a minor extent. Herein, a photoelectrochemical (PEC) detector based on the pseudo-binary MnIn_2_Se_4_ system is presented. A combination of optical measurements and DFT calculations confirmed that the nature of the bandgap in MnIn_2_Se_4_ is indirect. Its performance outclasses that of parent compounds, reaching responsivity values of 8.41 mA W^−1^. The role of the non-centrosymmetric crystal structure is briefly discussed as a possible cause of improved charge separation of the photogenerated charge carriers.

## Abstract 2

The lack of centrosymmetry in MnIn_2_Se_4_ crystals can lead to improved charge separation and enhanced responsivity of the photodetector prepared therefrom.

## Graphical abstract

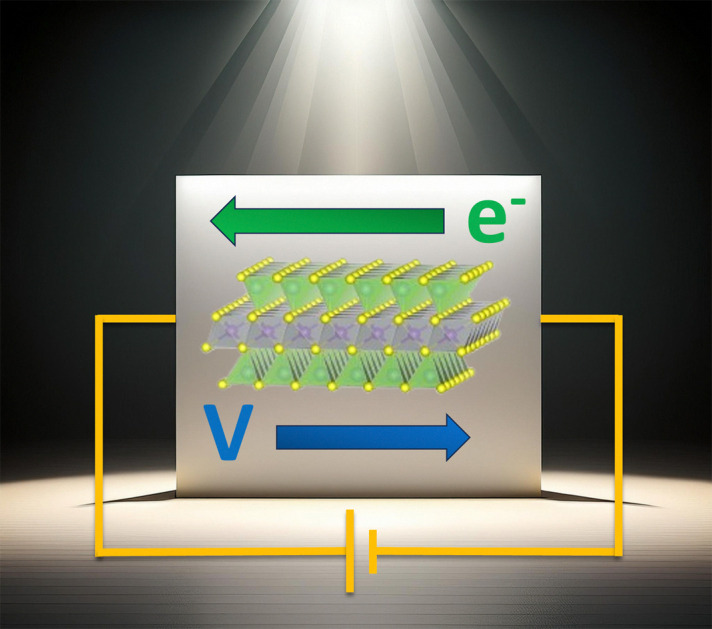

## 1. Introduction

Nanostructured two-dimensional (2D) materials play a leading role in modern chemistry due to their unique structure characterized by features such as extended exposed surfaces, anisotropic chemical bonding patterns, and short charge carrier diffusion length, which endow them with superior properties in the (opto)electronic,^1^ spintronics,^2^ and catalytic fields.^3^ 2D transition metal chalcogenides (TMCs, *e.g.* MoS_2_ and WS_2_) paved the way for a wide class of novel electronic nanodevices thanks to their intrinsic bandgap^4^ associated with their van der Waals structure,^5^ which allows the synthesis of few-layer crystals. These crystals have been implemented as core materials in various nanostructured optoelectronic devices, such as photodetectors, transistors, and pseudocapacitors.^6–10^ As an effect of quantum confinement and surface plasmon resonance, the reduced dimensionality of 2D TMCs introduces several size-dependent thermal^11^ and optical properties^12,13^ as shown in the case of metallic PtSe_2_ which presents semiconducting properties when its size is scaled-down to a few layers.^14^

2D TMCs have witnessed explosive development for various applications in the last few decades. Among them, binary monochalcogenide, dichalcogenide, and trichalcogenide (*e.g.*, GaSe,^15^ WS_2_,^16^ and In_2_S_3_^17^) photoresponsive devices have been thoroughly investigated. The study of ternary 2D chalcogenide materials has recently attracted growing interest due to their peculiar features which further expand the chemistry of chalcogenide materials,^18,19^ introducing multiple degrees of freedom in tailoring their composition to the benefit of phase engineering,^20,21^ and bandgap modulation.^22^ A plethora of structures can be described by the general formula of A_*x*_B_*y*_X_*z*_ with spacing from 1D-, 2D- to 3D-structures.^23,24^ In the case of A^II^B^III^_2_X^VI^_4_ chalcogenides, the relationship between the number of octahedral and tetrahedral sites is a key factor in determining the crystal structure of the resulting materials.^25^ When octahedral (Ω) or tetrahedral (τ) coordination prevails, a 3D spinel or tetragonal structure is formed, respectively. If a (1(Ω) : 2(τ)) balance is observed, the system holds a layered rhombohedral ZnIn_2_S_4_-type structure, described by the stacking of octahedral and tetrahedral sheets in an alternated sequence.^26^ The distribution of the cationic species within octahedral and tetrahedral sites can assume two configurations, named “normal” A^(Ω)^B^()^_2_X_4_; *e.g.* MgAl_2_Se_4_^27^ and “inverse” A^(τ)^B^(Ω)^, B^(τ)^X_4_*e.g.* ZnIn_2_S_4_^28^ rhombohedral structures. Besides ordered crystal structures, partially disordered and pseudo-binary alloyed phases can be synthesized. The disorder of the cations within different crystalline sites is described using the normality index *λ*, which describes the fraction of metallic atoms located in their respective lattice. When the index *λ* = 1/3 a stochastic distribution is reached and a pseudo-binary alloyed material is obtained,^29^ as shown in the cases of MgIn_2_S_4_ and MnIn_2_Se_4_.^30,31^

The diffusion of cationic species within octahedral and tetrahedral positions can be induced *via* vacancy-controlled processes as an effect of the temperature (*T* > *T*_critical_) during the crystal growth,^32^ or by high-pressure post-treatments.^33^ As a consequence, the structural and electronic properties of these materials might present a strong dependence on both growing temperature and quenching rates, which determine the presence of kinetic ion distribution in the final material. The order/disorder transitions are known to affect the physical properties of semiconducting materials to varying extents, ranging from the introduction of additional states in the bandgap,^34^ to the alteration of the electronic structure of the resulting disordered phase,^35^ suggesting potential effects in the optoelectronic devices prepared thereof. Significant attempts have been devoted to the study, design, and synthesis of various photocatalytic systems based on the “inverse” rhombohedral phases.^36,37^ ZnIn_2_S_4_ has been employed as a photocatalyst for dye degradation,^38,39^ hydrogen production,^40,41^ CO_2_ reduction^42,43^ N_2_ fixation,^44^ and light-driven synthetic reactions,^45^ either in the presence of noble metal nanoparticles or as a part of a heterojunction system. In addition, we have recently shown that ZnIn_2_S_4_ crystals can be used to fabricate visible light-NIR photodetectors characterized by high responsivity, small dark current, and a switching ratio up to 10^−6^.^46^

In contrast, “normal” and “disordered” pseudo-binary rhombohedral A^II^B^III^_2_X^VI^_4_ structures have been investigated to a minor extent.^47^ Non-intentionally doped MnIn_2_Se_4_ is a pseudo-binary layered n-type semiconductor^48^ that can be prepared *via* chemical vapor transport (CVT) or wet chemistry techniques. Despite being characterized by a narrow band-gap (*E*_g_ 1.55–1.85 eV) and wide visible light absorption,^49^ the study of photoelectronic MnIn_2_Se_4_ properties has received only little attention so far.^27,50,51^

MnIn_2_Se_4_ belongs to the non-centrosymmetric *R*3*m* space group. The lack of centrosymmetry in semiconductor materials produces a polarization electric field along the crystal, with different surfaces characterized by a prevalence of positive or negative charge. This field has been recognized as a driving force able to assist charge separation processes of photogenerated electrons and holes within the crystal, which represents the major kinetic drawback in semiconductor photocatalysis.^52,53^ Therefore, the non-centrosymmetric 2D MnIn_2_S_4_ appears as an attractive candidate to attain superior photoelectronic catalytic performances in the context of ternary chalcogenide materials. MnIn_2_Se_4_ nanosheets prepared *via* solvothermal synthesis have been employed as a photocatalyst for water-splitting reactions under UV illumination, in the presence of supported CoSeO_3_ nanoparticles as a co-catalyst. Interestingly, the superior photocatalytic properties of porous 2D MnIn_2_Se_4_ crystals allow hydrogen production even in the absence of sacrificial reagents, which normally hinder the practical application of this class of photocatalyst. Moreover, the catalyst is characterized by excellent stability, as shown by the linear production of hydrogen over an extended period.^54^ However, solvothermal synthesis presents major drawbacks from the perspective of green chemistry, such as the use of solvents, expensive precursors, reducing agents, and the presence of byproducts that require further purification processes. Consequently, it results in an inefficient atom economy.^55^ High-quality few-layered nanocrystals can also be prepared by mechanical exfoliation of bulk crystals^56^ prepared using a CVT technique with a stoichiometric mixture of the elements as the starting material in the presence of a catalytic amount of transporting agent (*e.g.*, iodine,^57^ or AlCl_3_^31^). Mechanically exfoliated few-layer MnIn_2_Se_4_ nanocrystals have been employed in field-effect transistors (FETs) showing excellent optoelectronic properties. The transfer characteristic of MnIn_2_Se_4_-based FETs under 532 nm laser irradiation shows an enhancement of the on–off ratio from 450 times to 1000 times in the range of gate sweep voltage from −40 to 40 V at a 10 V source–drain voltage.^57^ High photoresponse, electrical transport properties, and stability are important requisites in the design of efficient photodetector systems. In light of these considerations, we describe in this manuscript the photoelectric properties of exfoliated 2D MnIn_2_Se_4_ crystals supported on ITO glass as a photodetector system, where we observed a substantially high photocurrent density at 420 and 460 nm wavelengths (purple and blue, respectively) with high responsivity and stability, paving the way for expanding the research on novel ternary chalcogenides.

## 2. Results and discussion

**Fig. 1:**
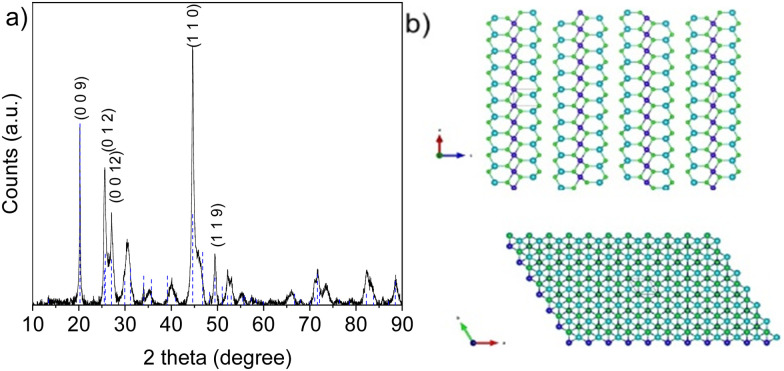
(a) XRD pattern of MnIn_2_Se_4_. (b) Crystal structure of rhombohedral MnIn_2_Se_4_ along the *c*-axis (side view) and *a*–*b* plane (top view). Blue atoms correspond to In, purple to Mn, and green to Se.

MnIn_2_Se_4_ crystals prepared *via* the CVT technique were characterized by X-ray diffraction (XRD), Raman, and UV-Vis Spectroscopy. The XRD pattern (Fig. 1(a)) shows the characteristic peaks of the non-centrosymmetric rhombohedral *R*3̄*m* phase reported by Döll *et al.*^31^ with lattice parameters of *a*, *b* = 4.057 Å and *c* = 39.497 Å (PDF: 00-046-1050). The crystal structure is depicted in Fig. 1(b). The unit cell is composed of a sequence of three Se–M^(τ)^–Se–M^(Ω)^–Se–M^(τ)^–Se crystalline layers held together by van der Waals forces. The layers are formed by a combination of MnSe_6_ octahedral sheets sandwiched between two InSe_4_ tetrahedral layers coordinated in the axial positions.

**Fig. 2:**
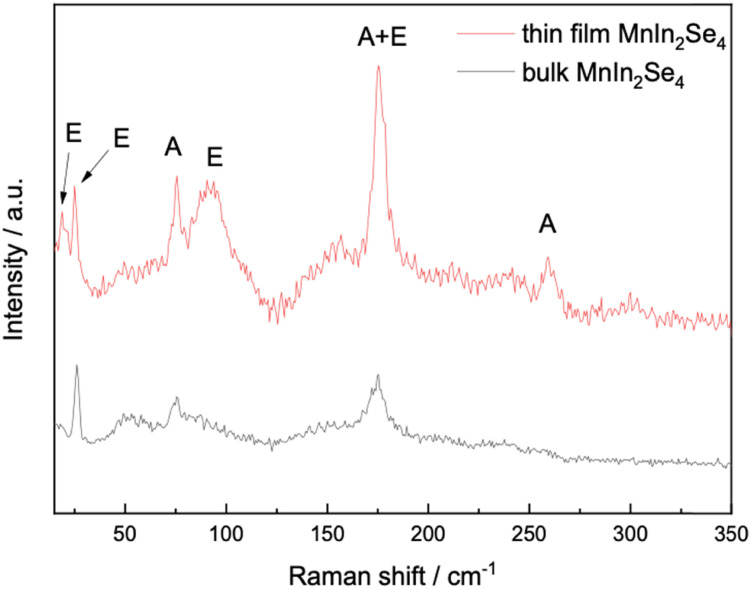
Raman spectra of bulk (black) and 2D (red) MnIn_2_Se_4_.

To the best of our knowledge, Raman spectroscopy analysis of MnIn_2_Se_4_ has not been reported in the literature. Thus, herein we report for the first time the vibrational modes of the material through a combination of experimentally acquired data with DFT simulations. For bulk MnIn_2_Se_4_, three peaks were observed at 26, 75, and 175 cm^−1^ while upon exfoliation of the material, three more peaks emerged at 19, 92, and 259 cm^−1^ (Fig. 2).

The theoretical calculations for the identification of the Raman modes of MnIn_2_Se_4_ are in excellent agreement with the experimental data (Fig. S1, ESI†). The peaks at 75 and 259 cm^−1^ are the vibrational frequencies related to the A vibrational mode. The peak at 175 cm^−1^ contains both vibrational modes A and E. We also confirm that there are vibrational modes A with vibrational frequencies at 184.6, 238.7, 252.1, and 256.5 cm^−1^ with zero Raman intensity in the bulk phase. The phonon modes corresponding to the most prominent Raman peaks are presented in Fig. S2 (ESI†). These modes are grouped into five frequency ranges: 6.5, 21, 62, 86, and 165 cm^−1^. The angular orientations of the phonon modes within each group were analyzed. Phonon modes around 165 cm^−1^ are predominantly perpendicular to the 2D surface (5 out of 9 phonon modes). In contrast, those around 21 and 62 cm^−1^ are primarily parallel to the 2D surface (2 out of 6 phonon modes and 1 out of 3 phonon modes respectively). This observation aligns well with experimental results, which show a significant enhancement of the Raman peak near 165 cm^−1^ when transitioning from bulk to 2D, while the peaks around 26 and 75 cm^−1^ exhibit relatively minor intensity changes. However, we should mention that, as not all phonon modes within a given frequency range are strictly perpendicular or parallel to the 2D surface, it is not possible to categorize them exclusively into these two orientations. Nonetheless, all Raman peaks display varying degrees of intensity enhancement when transitioning from bulk to 2D.

The other Raman peaks are all assigned to the E phonon modes. As evident from the experimentally obtained data, upon exfoliation and reduction in the number of layers, in addition to the emergence of new peaks, the modes attributed to the A vibrational modes are amplified in intensity.

**Fig. 3:**
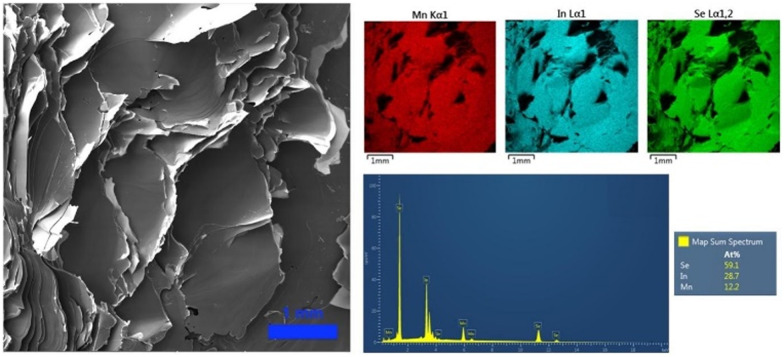
SEM image of a MnIn_2_Se_4_ crystal prepared *via* a CVT technique and the corresponding EDS elemental mapping, spectrum, and semi-quantitative analysis.

To further confirm the layered nature of the crystals synthesized *via* the CVT technique, their morphology was characterized by scanning electron microscopy coupled with energy dispersive spectroscopy (SEM/EDS). As displayed in Fig. 3, the SEM images represent the typical feature of layered materials, allowing us to identify the crystalline plane produced during the mechanical exfoliation. Furthermore, the EDS analysis revealed the homogenous distribution of the elements across the materials and an elemental quantification analysis in agreement with the expected stoichiometry of 1 : 2 : 4 for MnIn_2_Se_4_.

**Fig. 4:**
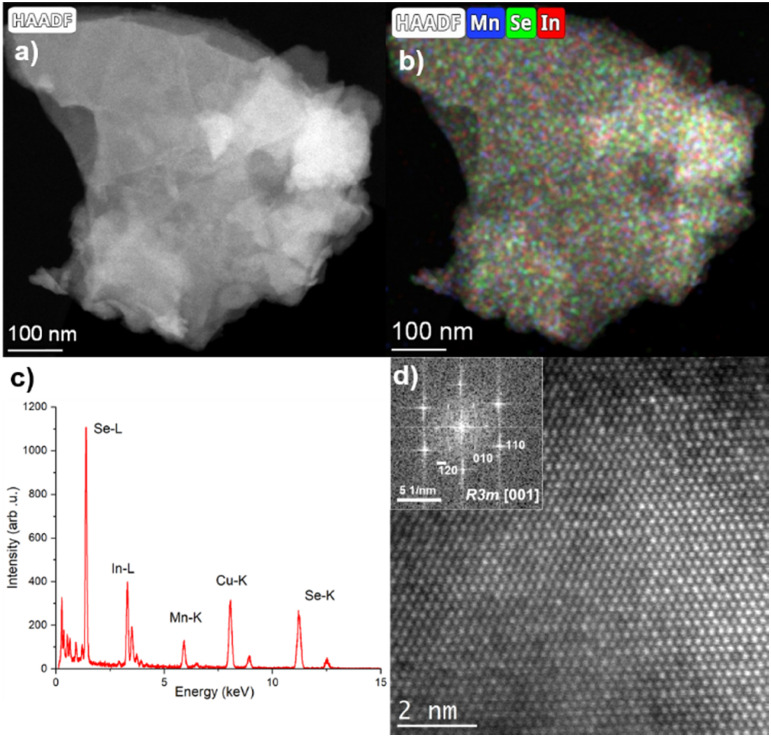
(a) Low magnification STEM-HAADF image of a MnIn_2_Se_4_ flake. (b) Corresponding Mn, Se, and In EDS maps, and (c) EDS spectrum extracted from the flake shown in (a). (d) HR-STEM HAADF image of the same flake. The inset displays the corresponding FFT pattern which has been successfully indexed with the MnIn_2_Se_4_*R*3*m* crystal structure seen along the [0 0 1] zone axis.

Fig. 4 shows the STEM analyses performed on the MnIn_2_Se_4_ flakes. The size of the flakes is typically between 100 and 400 nm. The elemental quantification was extracted from the EDS analyses and is shown in Table S1 in the ESI.† The composition is in excellent agreement with the SEM/EDS analysis, as shown above, and corresponds to the expected stoichiometry. According to the EDS maps (Fig. 4(b)) as well as medium (Fig. S3, ESI†) and high-resolution STEM-HAADF images, the flakes appear homogeneous in composition. Fig. 4(d) shows the HR-STEM HAADF micrograph acquired on the same flake. The high-crystalline quality of the flake is clearly presented. The corresponding FFT pattern was successfully indexed, matching the MnIn_2_Se_4_*R*3*m* crystal structure, seen along the [0 0 1] zone axis. It should be noted that all the analyzed FFT and SAED patterns (Fig. S4, ESI†) were successfully indexed by using the expected crystal structure. All these results highlight the high-crystalline quality of the synthesized material.

**Fig. 5:**
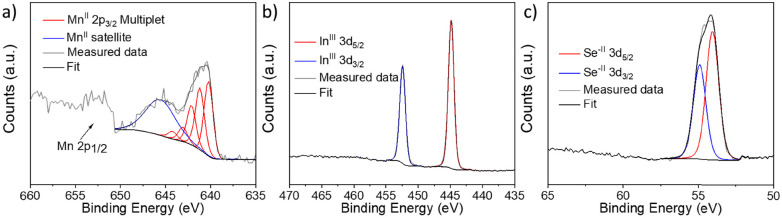
High-resolution XPS spectra of (a) Mn 2p, (b) In 3d, and (c) Se 3d regions.

**Fig. 6:**
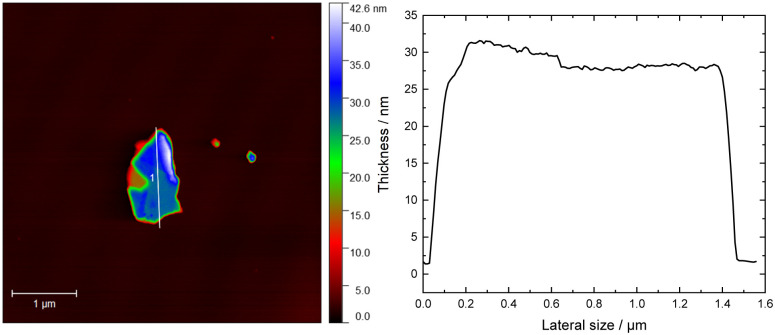
AFM image and the corresponding height profile plotted for an exfoliated MnIn_2_Se_4_ flake.

Surface oxidation states were analyzed by X-ray photoelectron spectroscopy (XPS). The composition ratio calculated from the wide-survey spectrum was 1 : 2.05 : 3.78 (Fig. S5, ESI†) which is very close to the expected stoichiometry. Notably, the oxygen concentration was very low at *ca.* 3.8% showcasing good stability in ambient conditions. The core-level spectra of In 3d and Se 3d only displayed one oxidation state with energies similar to those reported previously.^57^ Furthermore, the high-resolution XPS spectrum of In 3d shows two clear, well-defined peaks corresponding to In 3d_5/2_ and 3d_3/2_ with a 7.54 eV spin–orbit separation (Fig. 5(b)), while lastly, the Se 3d high-resolution XPS peak was deconvoluted into Se 3d_5/2_ and Se 3d_3/2_ with a spin–orbit splitting of 0.86 eV, ascribed to Se^−II^ (Fig. 5(c)). On the other hand, the spectrum of Mn 2p exhibited a much more complex structure (Fig. 5(a)). Previous studies on MnIn_2_Se_4_ in the literature show similar spectral features, where a broad main region from 639 eV to 645 eV is observed, accompanied by a strong satellite feature around 646 eV.^57,58^ In these studies, the presence of Mn^III^ or Mn^IV^ is reported. However, extensive studies on Mn-based compounds (such as oxides, hydroxides, and oxides-hydroxides) suggest that a more complex multiplet fitting approach may be more suitable for Mn deconvolution. This complexity is reinforced by the fact that Mn^II^, Mn^III^, and Mn^IV^ all exhibit similar binding energies which is in strong contrast to the aforementioned reports.^59,60^ Notably, only Mn^II^ species are commonly associated with satellite features, which aligns with the findings here.^59^ For Mn 2p deconvolution, we followed the procedure outlined in previous work,^59^ where the peak intensity ratios were adjusted slightly to obtain a satisfactory fit, acknowledging that values reported for Mn oxides may differ for other compounds. Due to the complexity of the deconvolution, these results should be considered to have qualitative significance at best and be interpreted with caution. The MnIn_2_Se_4_ flakes were ground and exfoliated by ultrasonication for 3 h with a programmed pulse of 2 s (*E*_n_ = 310 kJ) and DMF as a solvent. The suspension as prepared was analyzed by AFM (Fig. 6). The illustration of a representative AFM image of a MnIn_2_Se_4_ flake revealed a lateral size of 1.4 μm with a thickness of 30 nm. Further AFM images are shown in Fig. S6 (ESI†), where the thickness of the flakes varies between 20–50 nm and the lateral size between 1–1.6 μm.

**Fig. 7:**
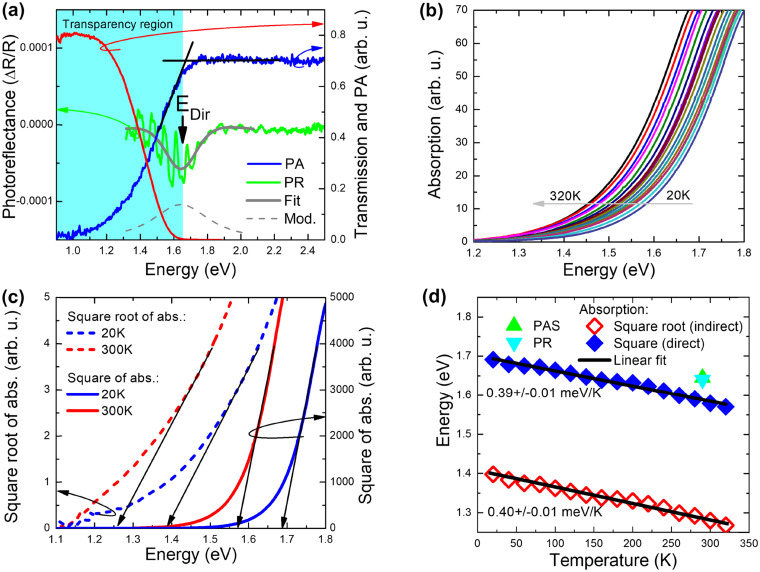
(a) Comparison of photoreflectance (green line), transmission (red line), and photoacoustic (blue line) spectra measured at room temperature. (b) Temperature dependence of absorption spectra. (c) Analysis of absorption spectra at low and room temperature. (d) Temperature dependence of indirect (open red diamonds) and direct (solid blue diamonds) gap determined from absorption measurements together with the direct gap determined at room temperature by photoacoustic spectroscopy (solid green triangle) and photoreflectance (solid cyan triangle).

To investigate the light-harvesting properties of the as-prepared suspension, absorbance spectra of the MnIn_2_Se_4_ nanosheets in DMF were recorded. As shown in Fig. S7a (ESI†), the exfoliated MnIn_2_Se_4_ material presents a broad absorption profile in the visible range characterized by a peak around 450 nm, highlighting the possibility of obtaining an active photodetector system in the zone of blue light. The corresponding Tauc plot diagram (Fig. S7b, ESI†) locates the optical band gap at 1.88 eV. This value is close to those reported in the literature for bulk MnIn_2_Se_4_ semiconductor materials, which typically lie between 1.55 and 1.85 eV.^49,50^Fig. 7 shows a comparison of photoreflectance (PR), transmission (T), and photoacoustic (PA) spectra measured at room temperature for a 10 μm thick MnIn_2_Se_4_ crystal. The transparency region for this sample reaches ∼1.6 eV. Above this energy the PA spectrum saturates. Using the knee method for the PA spectrum,^61,62^ it is possible to determine the energy gap from the intersection of the saturated area and the PA signal growth area, see the intersection of the black lines. It is worth noting here that the absorption begins well below 1.6 eV, and this is clear from both transmission and photoacoustic measurements. In order to determine the nature of this absorption (indirect *vs.* direct), these spectra are compared with the photoreflectance spectrum, which is not sensitive to indirect absorption. This method probes only direct optical transitions at singularities of the total optical density of states.^63^ A distinct resonance is observed in the PR spectrum at 1.64 eV and is attributed to the direct band gap in MnIn_2_Se_4_. This resonance is modulated by a Fabry–Perot oscillation, which is strong in the transparency region and disappears above the transparency region. In order to extract the energy of the direct gap in MnIn_2_Se_4_, the PR spectrum is fitted (see the solid grey line) by the Aspnes formula^64^ (eqn (1))1
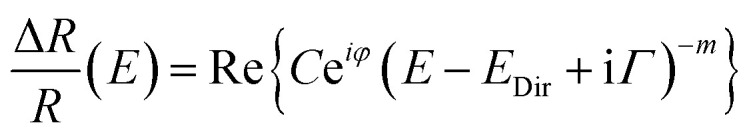
where Re denotes the real part of the complex function given in curly brackets, *E* is the photon energy, *E*_Dir_, *C*, *Γ*, and *φ*, are the energy, amplitude, broadening, and phase of PR resonance, respectively. *m* depends on the type of optical transition and for a direct band-to-band transition *m* = 2.5 is assumed.^64^ In addition, the modulus (Δ*ρ*) of the PR resonance is plotted for better visualization of the optical transition, see the dashed grey line in Fig. 7(a). This modulus for the complex function in curly brackets in eqn (1) is given by eqn (2).2
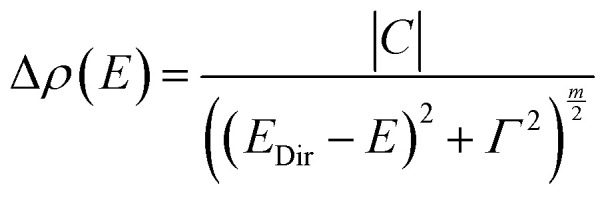


Thus, from this direct comparison of the PR spectrum with the transmission spectrum, it can be concluded that the fundamental energy gap in MnIn_2_Se_4_ is indirect. The conclusion is further supported by theoretical calculations which are presented in more detail below.

To determine the temperature dependence of the fundamental energy gap for MnIn_2_Se_4_, absorption measurements were carried out as a function of temperature, and the spectra are presented in Fig. 7(b). To determine the indirect gap, the square root of the absorption spectrum was drawn, where the linear dependence was extrapolated to zero and thus the indirect energy gap was determined. Additionally, the absorption square was plotted, and by linear extrapolation of this spectrum to zero the direct band gap was estimated (see the example analysis for determining the indirect and direct band gap in Fig. 7(c)). As can be seen, the direct energy gap determined in this way is ∼50 meV smaller than the energy gap determined using the photoreflectance method (compare the solid diamonds with the triangles in Fig. 7(d)). This difference is related to the non-parabolic nature of the valence band (note here that the linear relationship of the square of absorption used to determine the direct gap is expected for parabolic bands) and the sample thickness being too large to determine the direct band gap from absorption measurements.

Based on absorption measurements, it was found that the energy gap narrows with increasing temperature linearly with a factor of 0.39 ± 0.01 and 0.40 ± 0.01 μeV K^−1^ for the direct and indirect energy gap, respectively, see Fig. 7(d). This gives a narrowing of the gap by ∼110 meV in the temperature range of 20–320 K, which is slightly larger than that observed for group III–V semiconductors in the same temperature range (70–90 meV).^65^

As in other semiconductors, the narrowing of the energy gap with increasing temperature is associated with an increase in lattice parameters and electron–phonon interactions. Typically, the temperature dependence of the band gap is described using the empirical Varshni relationship^66^ (eqn (3)) or the Bose–Einstein formula^67,68^ (eqn (4)), which takes into account the electron–phonon interaction. The former relationship is given by:3
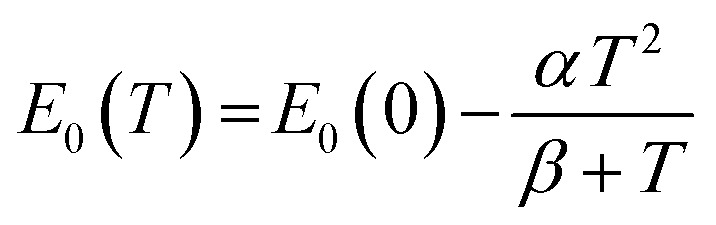
where *E*_0_(0) is the energy of transition at *T* = 0 K, whereas *α* and *β* are the so-called empirical Varshni coefficients, the values of which are typical for a given material but have no deeper physical meaning.

The latter formula, *i.e.*, the Bose–Einstein one, can be written in the form of:4
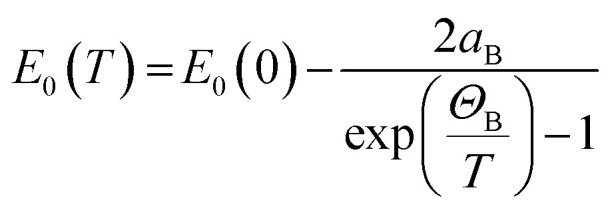
where *a*_B_ is the strength of the electron-average phonon interaction and *Θ*_B_ is the average phonon temperature. Both Varshni and Bose–Einstein relationships are non-linear in the low-temperature regime but it is easy to see that the Varshni relationship becomes linear *E*_0_(*T*) ≈ *E*_0_(0) − *αT* at *T* ≫ *β* and a linear relationship 
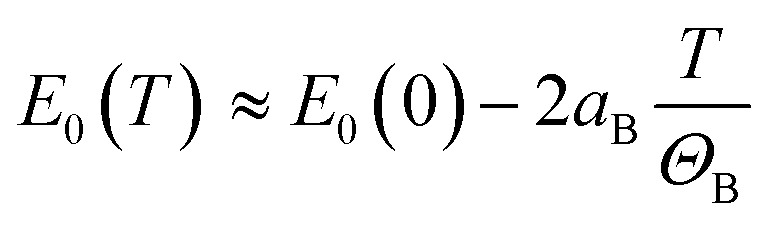
 can be assumed for the Bose–Einstein relationship at *T* ≫ *Θ*_B_. Therefore, the linear fitting of the experimental results for direct and indirect energy gaps is justified in this case, especially since we expect a magnetic phase for this crystal at low temperatures. Hence, the expected nonlinearity in this temperature range can be suppressed. It has recently been shown that the energy gap for CrSBr in the antiferromagnetic and paramagnetic phases exhibits a slightly different temperature dependence^69^ and therefore the description of energy gap dependence by the simplest formula, *i.e.* linear approximation, may be the best solution in the first approximation.

**Fig. 8:**
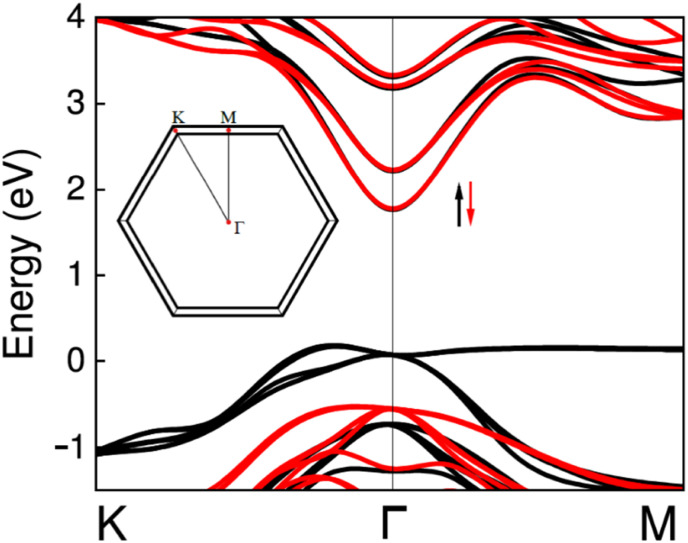
Electronic band structure for the *K*–*Γ*–*M* path for ferromagnet MnIn_2_Se_4_ calculated with a PBE0 hybrid functional.

Generally, MnIn_2_Se_4_ is a magnetic material due to the presence of Mn atoms, but the ferromagnetic phase is expected at very low temperatures. In our optical experiments, we are dealing with a paramagnetic phase, but this phase is a challenge in density functional theory (DFT) calculations. Therefore, we performed band structure calculations for the ferromagnetic phases using a hybrid functional (Fig. 8). So far, we have observed experimentally that the electronic structure (*i.e.*, the energy gap, *etc.*) does not change much when the crystal goes from the magnetic to the paramagnetic phase.^69^ This means that calculations for the ferromagnetic phase can be used to interpret optical transitions observed for the paramagnetic phase. For the ferromagnetic phase of MnIn_2_Se_4_, it is clearly visible that we are dealing with a crystal with a fundamental indirect gap. A direct gap in this crystal is at the Γ point of BZ and is larger by ∼110 meV than the indirect gap. Therefore, we claim that our DFT calculations support experimental data on the indirect nature of the band gap in MnIn_2_Se_4_.

### 2.1 Photoelectrochemical (PEC) photodetectors

**Fig. 9:**
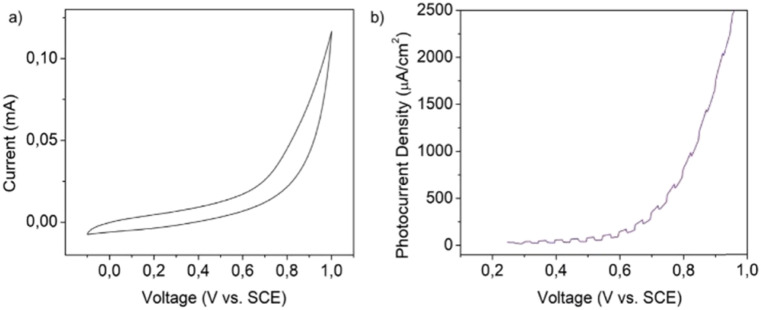
(a) Cyclic voltammetry of MnIn_2_Se_4_ supported on a GCE. Scan rate 0.1 V s^−1^. (b) Linear sweep voltammetry of a MnIn_2_Se_4_/ITO electrode.

Prior to the photoelectrochemical (PEC) experiments, the stability of the material was tested by submitting crystalline powder supported on a glassy carbon electrode (GCE) to potentials ranging from −0.1 to 1 V *vs.* SCE. As shown in Fig. 9(a), the material does not present any redox peak indicating the absence of any electrochemical reaction occurring in the specific potential window.

20 μL of the exfoliated MnIn_2_Se_4_ DMF suspension was dropcast onto an ITO glass equipped with a mask which set the active area to a spot of 0.196 cm^2^. The electrodes were tested in a PEC cell equipped with a saturated calomel electrode (SCE) and platinum wire as the reference and counter electrodes, respectively. The scheme of the experimental setup employed in the PEC tests is represented in Fig. S8 (ESI†). The photoresponsive behavior of the MnIn_2_Se_4_/ITO electrode was studied by linear sweep voltammetry (LSV) as depicted in Fig. 9(b). The photodetector under illumination with chopped purple light (power 100 mW) shows a photoresponsive behavior in the range of 0.25–1 V *vs.* SCE.

**Fig. 10:**
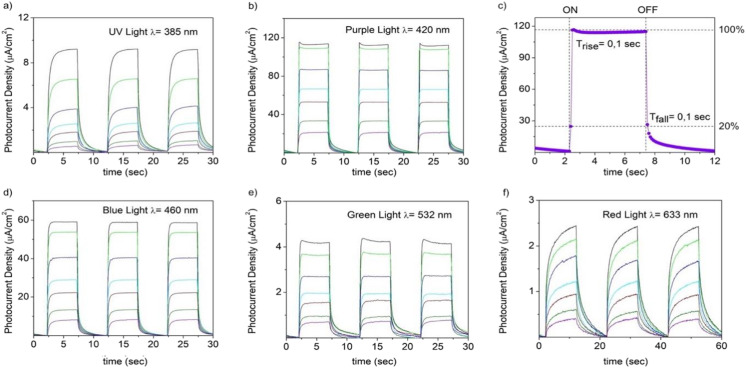
Power dependence of the photocurrent density under the illumination for a PEC-type MnIn_2_Se_4_/ITO photodetector upon irradiation with (a) UV (*λ* = 385 nm), (b) purple (*λ* = 420 nm), (d) blue (*λ* = 460 nm), (e) green light (*λ* = 532 nm), and (f) red light (*λ* = 633 nm) LED sources in 1 M KOH solution at 0.5 V *vs.* SCE. The power was set to the values of 50 mW (violet), 100 mW (olive), 200 mW (red), 300 mW (cyan), 500 mW (blue), 800 mW (green), and 1000 mW (black). (c) Response time of the PEC photodetector under the illumination with purple light LED with a power of 1000 mW.

A potential of 0.5 V *vs.* SCE was applied during the PEC tests to evaluate the photodetection performances of exfoliated MnIn_2_Se_4_ crystals supported on ITO toward LED light sources with wavelengths with frequencies spanning from the UV to the visible (purple light *λ* = 420 nm, blue light *λ* = 460 nm, green light *λ* = 532 nm, and red light *λ* = 633 nm). The chronoamperometric diagrams recorded with varying power of incident light are reported in Fig. 10.

As displayed in Fig. 10(a)–(f), the chronoamperometry diagrams recorded with varying the intensity of the incident light show a trend characterized by a continuous increase in the photocurrent variation (*I*_ph_ = *I*_light_ − *I*_dark_) with the increase of the power, as a consequence of the increased number of photons promoting the creation of electron–hole pairs within the semiconductor material. A comparison of the results obtained performing the illumination under the wavelengths of 385, 420, 460, and 532 nm (herein named for simplicity UV, purple, blue, and green light) in the presence of 1 M KOH as the electrolyte demonstrates a predominant response to purple light and blue light which determine a maximum photocurrent density of 112.8 μA cm^−2^ and ∼59 μA cm^−2^ respectively (power 1000 mW).

Beyond blue light, the photodetector demonstrates a weak response to UV, green, and red light, reaching the photocurrent's values of ∼9.2, ∼4.1, and ∼2.3 μA cm^−2^, respectively.

The response time for purple light is represented in Fig. 10(c). The photodetector demonstrates a fast response toward the illumination being characterized by a delay of 0.1 s in reaching the maximum current density and a recovery time of 0.1 s to diminish the current to 20% of the value assumed under irradiation.

**Fig. 11:**
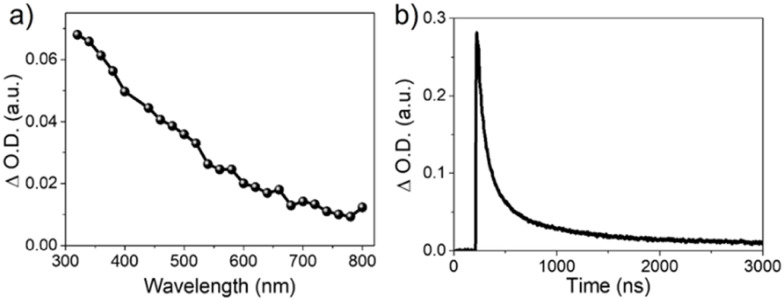
(a) Transient absorption spectrum of a N_2_-purged dispersion of MnIn_2_Se_4_ in acetonitrile, acquired at 500 ns upon 420 nm laser excitation. (b) Transient absorption decay of a N_2_-purged dispersion of MnIn_2_Se_4_ in acetonitrile, monitored at 360 nm upon 420 nm laser excitation.

In order to further investigate the excited state characteristics of the bi-dimensional MnIn_2_Se_4_, transient absorption (TA) experiments were carried out. The transient absorption spectrum of MnIn_2_Se_4_ has been acquired at 500 ns upon illumination with a laser pulse at a 420 nm excitation wavelength (Fig. 11). The TA spectrum of a MnIn_2_Se_4_ dispersion in acetonitrile under a N_2_ atmosphere shows a continuous band decreasing in intensity from the UV towards the NIR. This is in consistent with the band gap value of MnIn_2_Se_4_ (reported in the range of 1.55 to 1.85 eV and measured as 1.88 eV).

On the other hand, the transient decay of photogenerated species was monitored at 360 nm (Fig. 11(b)). The transient decay presents similar bimodal behavior in the range of ns–μs time scale, which can be fitted to a bi-exponential function (eqn (5)).5
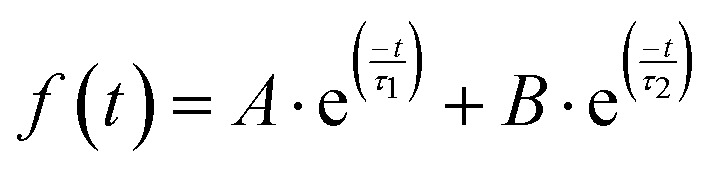


The fitting of the experimental data obtained from MnIn_2_Se_4_ dispersions in acetonitrile under a N_2_ atmosphere and monitored at 360 nm shows a fast component of *τ*_1_ = 79 ns and a second component of *τ*_2_ = 553 ns. These results indicate the presence of two different types of defects acting as charge carrier traps which are most likely positioned deep within the bandgap.

To characterize the performances of the photodetector, figures of merit such as responsivity and photoresponse have been determined for the MnIn_2_Se_4_/ITO system. The responsivity describes the relationship between the photogenerated current per unity of area and the power density of the incident light, as expressed by eqn (6):6
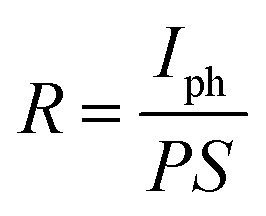
where the *I*_ph_ corresponds to the photocurrent obtained upon irradiation (*I*_ph_ = *I*_light_ − *I*_dark_), *P* indicates the power density and *S* is the effective area under illumination. The photoresponse expresses the relationship between *I*_ph_ and the current under dark as described by eqn (7):7
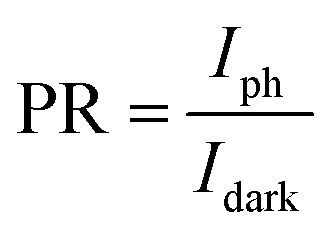


**Fig. 12:**
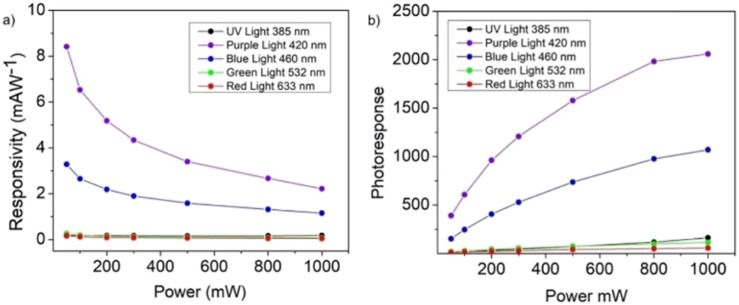
(a) Responsivity and (b) photoresponse for the PEC-type MnIn_2_Se_4_/ITO photodetector in 1 M KOH solution at 0.5 V *vs.* SCE.

The responsivity and the photoresponse diagrams for the MnIn_2_Se_4_/ITO photodetector for the case of 1 M KOH as the electrolyte solution are reported in Fig. 12.

The responsivity of the photodetector shows a trend identified by an exponential decrease of R in the increase with the power, owing to the higher fraction of electrons per unity of power that are promoted to the conduction band upon low-intensity illumination. Conversely, the responsivity diagram shows a minimum at high power values determined by a lower population of free charge carriers respective to the power of the incident light. In contrast, the photoresponse of the device demonstrates a steady growth concerning the power, which reflects the steady increase of the photogenerated current compared to the current under dark conditions.

The maximum values of responsivity and photoresponse are reached under purple and blue light irradiation, setting the maximum parameters for the MnIn_2_Se_4_/ITO photodetector to *R* = 8.41 mA W^−1^, PR = 2060 and *R* = 3.28 mA W^−1^, PR = 1070 respectively. The responsivity obtained under irradiation with UV, green, and red light demonstrates values an order of magnitude lower concerning purple and blue light pointing out a certain selectivity to the frequency in the range of 400 nm.

The values of responsivity obtained for MnIn_2_Se_4_/ITO can be compared with the results reported in the literature for PEC-type photodetectors performing in the presence of low applied potential (0–1 V). As shown in Table 1, the values obtained in the present work for the MnIn_2_Se_4_/ITO photodetector exceed those reported for other layered mono- and ternary-sulfides reaching the values obtained for perovskite-based heterojunction systems.

**Table 1:** Values of responsivity obtained for photodetector systems based on various materials

Material(s)	Device configuration	Electrolyte	Applied potential (V)	Responsivity (mA W^−1^)	Wavelength (nm)	Ref.
ZnIn_2_S_4_–Ag. Gel-Pt	Metal–semiconductor–metal	—	0	0.02	Simulated sunlight	70
InSe nanosheets	PEC-type	0.2 M KOH	1	3.3 × 10^−3^	455	71
				4.0 × 10^−3^		
Black phosphorous nanosheets	PEC-type	0.1 M KOH	0	1.9 × 10^−3^	Simulated sunlight	72
				2.2 × 10^−3^		
GeSe nanosheets	PEC-type	0.1 M KOH	0.3	0.044	Simulated sunlight	73
				0.076		
SnS	PEC-type	0.1 M Na_2_SO_4_	0.6	0.018	365	74
Perovskite (CH_3_NH_3_PbI_3_)	Metal–semiconductor–metal	—	5	4.4	633	75
Perovskite (CH_3_NH_3_PbI_3_) PDPP3T	Metal–semiconductor–metal	—	1	10.7	365	76
				25.5	650	
				5.5	937	
MnIn_2_Se_4_	PEC-type	1 M KOH	0.5	8.41	420	This work

**Fig. 13:**
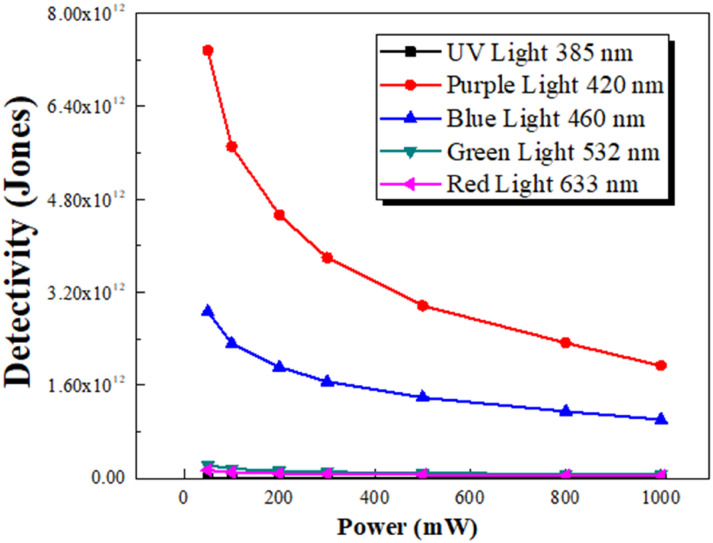
Detectivity (D*) of the MnIn_2_Se_4_ photodetector across various wavelengths.

Furthermore, detectivity (D*) is another crucial parameter that quantifies the efficiency of a photodetector in detecting weak optical signals. It is expressed in eqn (8):8
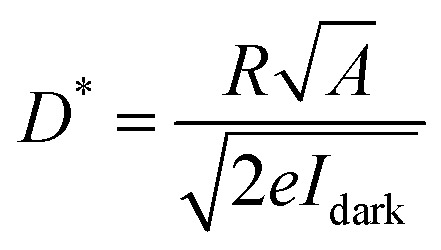
where *R* is responsivity, *A* is the area, and *e* is the electron charge. The detectivity plot displayed in Fig. 13 revealed a maximum D* of 7.40 × 10^12^ and 2.87 × 10^12^ Jones under 420 nm purple light and 460 nm blue light, respectively. The strong correlation between D* and the device's responsivity under specific wavelengths underscores the reliable photodetection capabilities of MnIn_2_Se_4_ in the visible spectrum. In addition, the noise equivalent power (NEP) represents the input signal power required to achieve a signal-to-noise ratio (S/R) of 1 within a 1 Hz output bandwidth. It is calculated using eqn (9):9
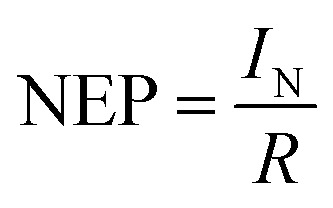
where *I*_N_ is the noise current, directly influenced by the dark current. A detailed comparison of NEP values for different wavelengths, presented in Table S2 (ESI†). Finally, the long-term stability of a photodetector plays a role of primary importance in the practical application of the device. To fulfill this requirement, long-time on–off cycle experiments have been performed on MnIn_2_Se_4_/ITO photodetectors. As shown in Fig. S9 (ESI†), the photodetector shows stable on/off behavior under blue light irradiation over an extended irradiation time, showing a minor decline of signal intensity (5%) which can be attributed to the poor cohesion of MnIn_2_Se_4_ nanosheets to the ITO surface.

## 3. Conclusion

To summarize, the properties of MnIn_2_Se_4_, a novel layered, ternary material, have been thoroughly characterized with respect to its structure, morphology, and optics using a combination of experimental results and DFT calculations. In this manuscript, we report the indirect nature of the material's bandgap and additionally, the material has been studied as a photodetector in an alkaline environment where it showed excellent response upon blue and purple irradiation with a photocurrent density maximum of 59 and 112.8 μA cm^−2^ respectively. The responsivity of the material at these wavelengths was found to be higher than that of similarly reported devices with values at 8.41 and 3.28 mA W^−1^ for 420 and 460 nm wavelength illumination. Based on these results, the investigation of the usage of ternary chalcogenides as photodetectors is highly anticipated.

## 4. Experimental section

### 4.1 MnIn_2_Se_4_ synthesis

MnIn_2_Se_4_ crystals were prepared by chemical vapor transport (CVT) in a quartz glass ampoule. Manganese (99.95%, −100 mesh, Mateck), indium (99.9999%, 1–2 mm, Wuhan Xinrong New Material Co., China) and selenium (99.9999%, 2–4 mm, Wuhan Xinrong New Material Co., China) were placed in quartz ampoule (35 × 180 mm, wall thickness 3 mm) in amounts corresponding to 20 g of MnIn_2_Se_4_ together with 0.5 g of iodine (99.9%, granules, Fisher Scientific, USA) and melt sealed under high vacuum (under 10^−3^ Pa, diffusion pump with LN_2_ trap). The ampoule was first placed in a muffle furnace and heated at 500 °C for 25 h, at 600 °C for 50 hours and at 800 °C for 50 hours. After the reaction in a muffle furnace, the ampoule was placed in a horizontal two-zone furnace for CVT crystal growth. First, the growth zone was heated at 850 °C and the source zone at 600 °C. After two days, it was thermal gradient reversed and the crystal growth took 10 days (source zone at 850 °C and growth zone at 750 °C). After growth, the ampoule was opened in an argon-filled glovebox.

Solvent-assisted exfoliation was performed in DMF by ultrasonication. First, the CVT growth crystals were ground in an agate mortar and sieved. Subsequently, ultrasonication was performed in DMF (1 mg mL^−1^) using a 100 W sonotrode under an argon atmosphere (Bandeline Sonoplus HD2200).

### 4.2 Material characterization techniques

XRD patterns were acquired on a Bruker D8 Discover powder diffractometer (Bruker, Germany) in Bragg–Brentano parafocusing geometry with Cu K_α_ radiation, in an angular range of 5–90° 2*θ*, with a step size of 0.020° and the data was analyzed with HighScore Plus 3.0e software. The Raman spectrum were obtained using an inVia Raman microscope (Renishaw, England) in backscattering geometry with a CCD detector for Raman spectroscopy. DPSS laser (532 nm, 50 mW) with an applied power of 5 mW and 50× magnification objective was used for the measurement. Instrument calibration was achieved with a silicon reference which gives a peak position at 520 cm^−1^ and a resolution of less than 1 cm^−1^. The samples were suspended in deionized water (1 mg mL^−1^) and ultrasonicated for 10 min. The suspension was deposited on a small piece of silicon wafer and dried.

The morphology was investigated using SEM with a FEG electron source (Tescan Lyra dual beam microscope). Elemental composition and mapping were performed using an EDS analyzer (X-Max^N^) with a 20 mm^2^ SDD detector (Oxford instruments) and AZtecEnergy software. To conduct the measurements, the samples were placed on carbon conductive tape. SEM and EDS measurements were carried out using a 10 kV electron beam. The STEM was performed with a Tescan Lyra dual beam microscope equipped with an FEG electron source and STEM sample holder. To conduct the measurements, the sample suspension was dropcast onto a 200 mesh Cu TEM grid and dried in a vacuum oven (50 °C). STEM measurements were carried out using a 30 kV electron beam.

Aberration-corrected high-resolution (scanning) transmission electron microscopy imaging (HR-(S)TEM) and energy-dispersive X-ray spectroscopy (EDS) were performed using a FEI Titan Cubed Themis microscope (University of Cádiz), which was operated at 80 kV. It is equipped with a double Cs aberration-corrector, a monochromator, an X-FEG gun, an ultrahigh-resolution energy filter (Gatan Quantum ERS), which allows working in dual-EELS mode, and a super-X EDS detector, which consists of four windowless SDD detectors that can be read out independently. HR-STEM imaging was performed using a high-angle annular dark-field (HAADF) detector. EDS quantification was achieved using the Brown–Powell model for the ionization cross sections. Selected Area Electron Diffraction (SAED) and fast Fourier transform (FFT) patterns were automatically indexed using the JEMS software.^77^

High-resolution XPS was performed using an ESCAProbeP spectrometer (Omicron Nanotechnology Ltd, Germany) with a monochromatic aluminum X-ray radiation source (1486.7 eV). Wide-scan surveys of all elements were performed, with subsequent high-resolution scans of manganese (Mn 2p), indium (In 3d), and selenium (Se 3d). Relative sensitivity factors were used to evaluate the carbon-to-oxygen (C/O) ratios from the survey spectra. The samples were placed on a conductive carrier made from a high-purity silver bar. An electron gun was used to eliminate sample charging during measurement (1–5 V). All XPS spectra were analyzed by CasaXPS software.

AFM observations were implemented using a Ntegra Spectra from NT-MDT. The surface scans were carried out in a tapping (semi-contact) mode. Cantilevers with a strain constant of 1.5 kN m^−1^ equipped with a standard Si tip with a curvature radius lower than 10 nm were used for the measurements. The AFM imaging was performed by drop-casting a sample suspension (1 mg mL^−1^) on freshly cleaved mica substrate. The measurements were conducted in ambient conditions with a scan rate of 1 Hz and scan line of 512. Data analysis was done using the software package Gwyddion.

### 4.3 Electrochemistry

The electrochemical characterization by means of cyclic voltammetry was performed using an Autolab PGSTAT 204 (Metroohm, Switzerland). All glassy carbon electrodes were cleaned by polishing with an alumina suspension to renew the electrode surface then washed and wiped dry prior to any use. For the measurement a modified glass carbon electrode was used as a working electrode, with a calomel reference electrode, and a platinum counter electrode.

ITO electrodes were cleaned by rinsing with water, ethanol, and acetone and dried at 60 °C. An adhesive mask was applied to reduce the active area to a circle of 0.5 cm in diameter. The samples were dispersed in DMF as the organic solvent to obtain a 1 mg mL^−1^ suspension. The suspension was then sonicated for 5 min at room temperature before every use. A cleaned ITO electrode was then modified by coating with a 10 μL aliquot of the suspension and left to dry in a box oven at 60 °C to prepare MnIn_2_Se_4_/ITO electrodes. The modified GC electrodes, SCE reference electrode, and platinum counter electrode were then placed into an electrochemical cell which contains the electrolyte solution, and the measurements were then taken. All measurements were performed for three consecutive scans at a scan rate of 100 mV s^−1^.

### 4.4 Optical measurements

For photoreflectance measurements, the sample was illuminated with a spectrum of white light from a halogen lamp, which, after being reflected from the sample, was directed at a monochromator (Triax 550) using appropriate lenses. The dispersed light was analyzed step by step (wavelength by wavelength) by measuring its intensity using a silicon photodiode. A 405 nm laser with a power of 80 mW was used to modulate the surface electric field in the sample. The laser beam was modulated at a frequency of 280 Hz. Changes in the reflection (Δ*R*) caused by laser modulation were measured in the lock-in technique using a phase-sensitive nanovoltmeter (Stanford SR830). The reflectance spectrum (*R*) was simultaneously measured to obtain the photoreflectance spectrum, *i.e.*, Δ*R*/*R*. The spot size of the probing beam (white light) and the size of the laser beam on the sample were very comparable and had a diameter of 1–2 mm. For more details of photoreflectance measurements see ref. 63.

For photoacoustic measurements, the sample was placed in a photoacoustic chamber equipped with a broadband microphone.^78^ The sample was illuminated with monochromatic light from a halogen lamp after passing through a monochromator (iHR 320). The measurements were performed using the lock-in technique with a phase-sensitive nanovoltmeter (Stanford SR830) and modulating the light beam with a frequency of 20 Hz. Powdered carbon was used to calibrate the system.

For transmission (absorption) measurements at various temperatures, the sample was placed in a closed-cycle helium-cooled cryostat, which allows measurements in the temperature range from 10 to 325 K. The light from the halogen lamp, after passing through the monochromator (Omni-λ300i), was directed at the sample, and after passing through the sample, at the silicon (and InGaAs) photodiode. The light beam was modulated in front of the monochromator at a frequency of 280 Hz and its transmission through the sample was measured in the lock-in technique with a Stanford SR830 phase-sensitive nanovoltmeter.

### 4.5 DFT calculations

The electronic structure and Raman spectra of MnIn_2_Se_4_ were computed by density functional theory (DFT) calculations using two codes: Quantum ESPRESSO code^79,80^ together with norm-conserving SG15 pseudopotentials^81,82^ and Vienna *Ab Initio* Simulation Package together with the PAW method.^83–86^ In the first approach, we calculated the Raman spectrum using the Perdew, Burke, and Ernzerhof (PBE) exchange–correlation functional.^87^ The cut-off energies of the wave function and charge density were chosen to be 70 Ry and 280 Ry respectively. All structures are relaxed until the force is less than 5.0 × 10^−4^ Ryd Bohr^−1^. We used an 8 × 8 × 1 *k*-mesh for surface structure calculation and Γ-point only for molecular structure calculations. Electronic spin-polarization is also considered in all the simulations, and we verified that for the bulk MnIn_2_Se_4_ structure, the ferromagnetic structure has a 1.47 eV lower energy per manganese atom than the anti-ferromagnetic ordering. Thus, we assumed a ferromagnetic state in all the following calculations. To overcome the lack of van der Waals interactions in the PBE functional, the semiempirical Grimme's DFT-D3 correction^88^ is applied. The resulting exfoliation energy is 13.54 meV Å^−2^, which also indicates that bulk MnIn_2_Se_4_ is easy to exfoliate and confirms its potential application as a low-dimensional material.^89^

As the PBE level of DFT predicts no band gap for bulk MnIn_2_Se_4_ a Hubbard *U* correction is applied,^90^ which improves the PBE electronic structure and correctly predicts the semiconducting nature of the material. In the MnIn_2_Se_4_ structure shown in Fig. S10 (ESI†), selenium atoms are divided into two types, namely the Se-outer and Se-inner. The outer selenium atoms only have chemical bonds with indium atoms and are placed on the outer side of the MnIn_2_Se_4_ layer. The inner selenium atoms have both chemical bonds with manganese atoms and indium atoms and are placed inside the MnIn_2_Se_4_ layer. In our treatment, we consider these two types of selenium atoms as different types. We therefore calculated from first-principles the *U* values by using the HP module of Quantum ESPRESSO, which uses density-functional perturbation theory to calculate the constrained linear-response matrices employed in the prediction of the Hubbard *U* parameters.^91,92^ All the *U*-values are calculated using a 2 × 2 × 1 *q*-mesh and are obtained from a single calculation, meaning no self-consistent procedure convergence of the Hubbard parameters was applied. The *ortho*-atomic manifolds were used in all the Hubbard calculations as orbital projectors.^93^ We obtain 5.06 eV for the manganese 3d orbital, 3.34 eV for the inner selenium 4p orbital, and 3.11 eV for the outer selenium 4p orbital. For indium atoms, as the 4d orbital is very close–shell, the occupation number on the 4d-orbital is not going to change significantly when a perturbation is performed. As a consequence, we do not apply any Hubbard correction to the 4d orbital of indium atoms. An 8 × 8 × 1 *k*-points mesh is used in all self-consistent electronic calculations and linear response Hubbard-*U* calculations.

The Raman spectra were computed by the AiiDA-vibroscopy package,^94^ which uses the AiiDA^95–97^ interactive infrastructure to automatically submit jobs on remote machines, while managing data and workflows locally. We used the finite electric field^98,99^ and the finite displacement^100^ methods to carry out the calculation of the Raman tensors and force constants matrix needed for the prediction of the Raman spectra. These techniques allow for the usage of any functional, hence are suitable for considering the Hubbard corrections. The Raman spectra were computed considering a powder phase, a 532 nm incoming laser wavelength, and a temperature of 300 K. Since experimental Raman spectra are acquired at room temperature, the material is found in a paramagnetic state, with zero total magnetization. For the Raman calculations only, we model the magnetic state of the bulk in an anti-ferromagnetic configuration, which can be considered as a very first approximation of paramagnetic ordering. The simple anti-ferromagnetic structure is found to be sufficient to reach good agreement with experimental data (Fig. S1, ESI†), thus is sufficient to carry out normal mode analysis in this material.

Apart from the calculation at the DFT+*U* level, we also performed band structure calculations by means of a hybrid functional *via* the VASP code, with the aim of obtaining better quantitative agreement with the experimental band gap. For geometry optimization, we used, as in the Quantum ESPRESSO simulations, the PBE functional together with D3 corrections. The resulting lattice constants *a* = 4.045 Å and *c* = 39.718 Å are in good agreement with the experiments (4.057 and 39.497 Å). The refined calculation of electronic properties was carried out by employing the PBE0 hybrid functional. In order to further improve agreement with optical measurements we fitted parameters that mix GGA and HF exchange in the PBE0 functional in such a way as to achieve an experimental direct band gap. The final mixing parameter was fixed at 0.33 (for PBE0 it is 0.25). All calculations were performed with an energy cutoff of 600 eV and *k*-point sampling of 10 × 10 × 1.

## Data availability

The datasets generated during and/or analyzed during the study are accessible *via* the Zenodo repository: https://doi.org/10.5281/zenodo.13827444.

## Conflicts of interest

The authors declare no conflicts of interest.

## Supplementary Material

TC-013-D4TC04380D-s001
