# Supplementary material for: A photodetector based on the non-centrosymmetric 2D pseudo-binary chalcogenide MnIn2Se4
Source: J Mater Chem C Mater. 2025 Jan 15;13(10):5356–69. doi: 10.1039/d4tc04380d (PMC11783042; doi:10.1039/d4tc04380d)
Supplement: TC-013-D4TC04380D-s001 [file TC-013-D4TC04380D-s001.pdf]

Supplementary Information (SI) for Journal of Materials Chemistry C.
This journal is © The Royal Society of Chemistry 2025

Supporting Information

Photodetector Based on the Non-Centrosymmetric 2D Pseudo-Binary Chalcogenide $MnIn_2Se_4$

*Marco Serra,[1,2,] Nikolas Antonatos,[1,3] Luc Lajaunie [4,5] Josep Albero,[6] Hermenegildo Garcia,[6] Mouyi Weng,[7] Lorenzo Bastonero,[8] Kalyan Jyoti Sarkar,[1] Rui Gusmão,[1] Jan Luxa,[1] Rafał Bartoszewicz,[3] Jakub Ziembicki,[3] Nicola Marzari,[7,8,9] Iva Plutnarová,[1] Robert Kudrawiec,[3] Zdenek Sofer[1]**

[1]Department of Inorganic Chemistry, University of Chemistry and Technology Prague, Technicka 5, 166 28 Prague 6, Czech Republic; zdenek.sofer@vscht.cz

[2]Current Address: Istituto Italiano di Tecnologia, Via Morego 30, 16163, Genova; marco.serra@unimore.it

[3]Department of Semiconductor Materials Engineering, Faculty of Fundamental Problems of Technology, Wrocław University of Science and Technology, Wybrzeże Wyspiańskiego 27, 50-370, Wrocław, Poland

[4]Departamento de Ciencia de los Materiales e Ingeniería Metalúrgica y Química Inorgánica, Facultad de Ciencias, Universidad de Cádiz, Campus Río San Pedro S/N, Puerto Real, 11510, Cádiz, Spain

[5]Instituto Universitario de Investigación de Microscopía Electrónica y Materiales (IMEYMAT), Facultad de Ciencias, Universidad de Cádiz, Campus Río San Pedro S/N, Puerto Real 11510, Cádiz, Spain

[6]Instituto de Tecnología Química, Universitat Politècnica de València- Consejo Superior de Investigaciones Científicas (UPV-CSIC), Universitat Politècnica de València, Avda. de los Naranjos s/n, 46022, Valencia (Spain)

[7]Theory and Simulation of Materials (THEOS), École Polytechnique Fédérale de Lausanne (EPFL), Lausanne, CH-1015 Lausanne, Switzerland

[8]U Bremen Excellence Chair, Bremen Center for Computational Materials Science, and MAPEX Center for Materials and Processes, University of Bremen, D-28359 Bremen, Germany

[9]Laboratory for Materials Simulations, Paul Scherrer Institut (PSI), 5232 Villigen, Switzerland

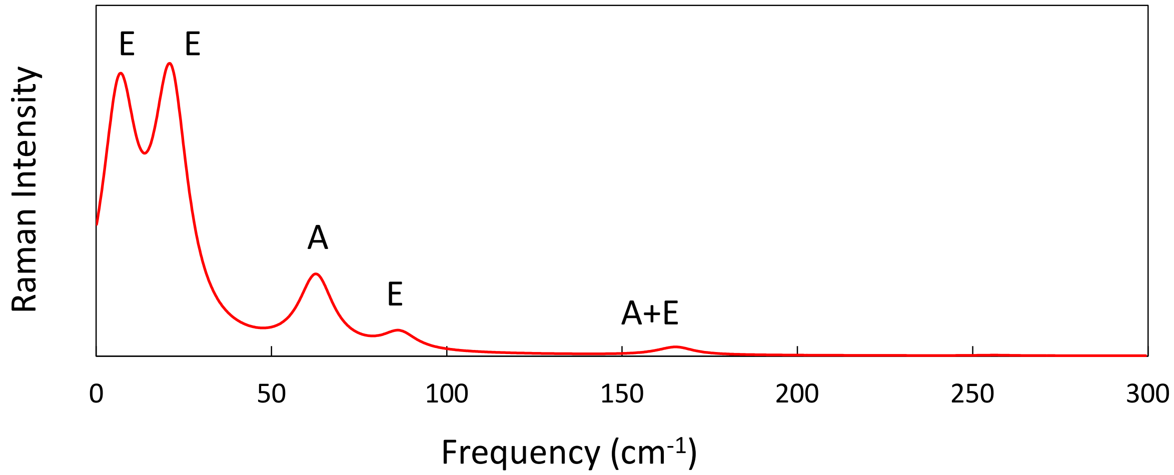

Figure S1. DFT calculated Raman spectra of bulk $MnIn_2Se_4$. The A peaks indicate the vibrational modes perpendicular to the 2D plane, while the E peaks indicate the vibrational modes parallel to the 2D plane. A+E indicates the normal mode including both parallel and perpendicular atomic motions.

a)

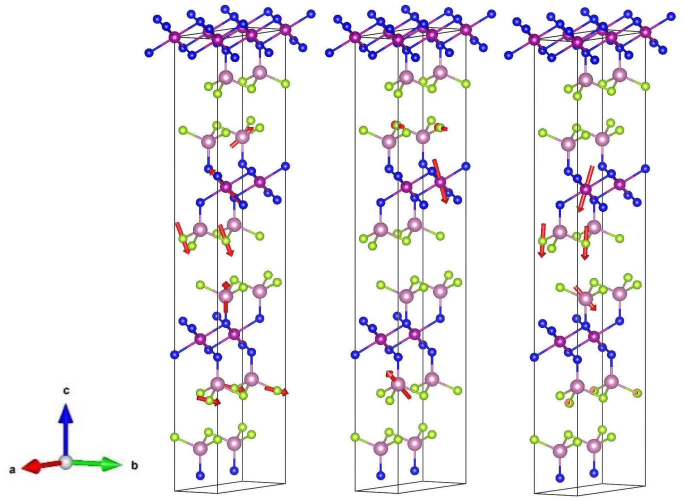

b)

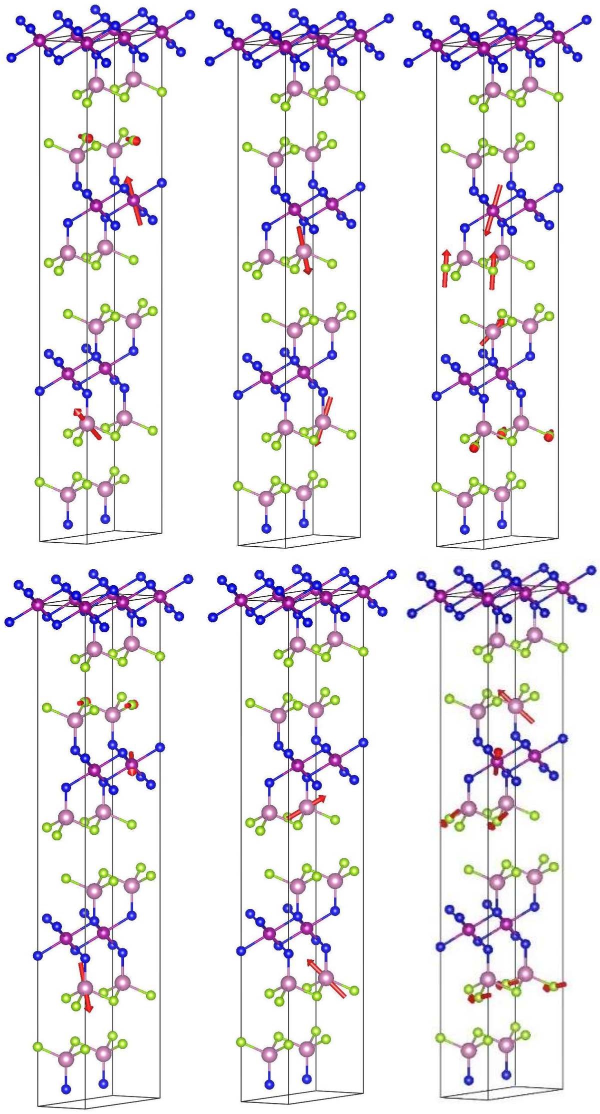

c)

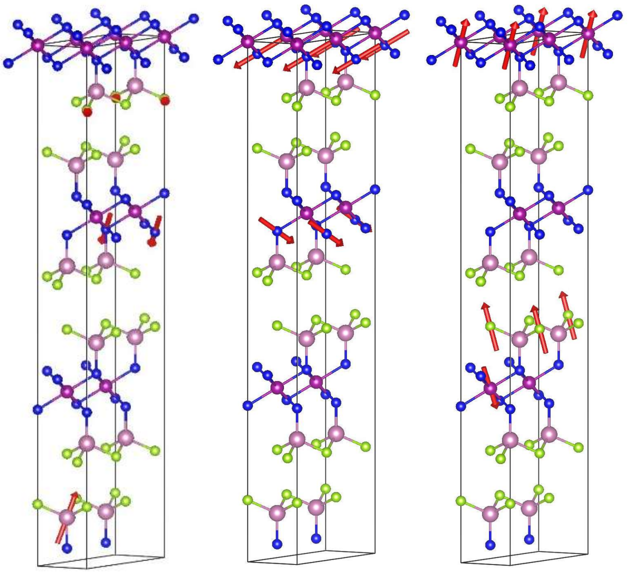

d)

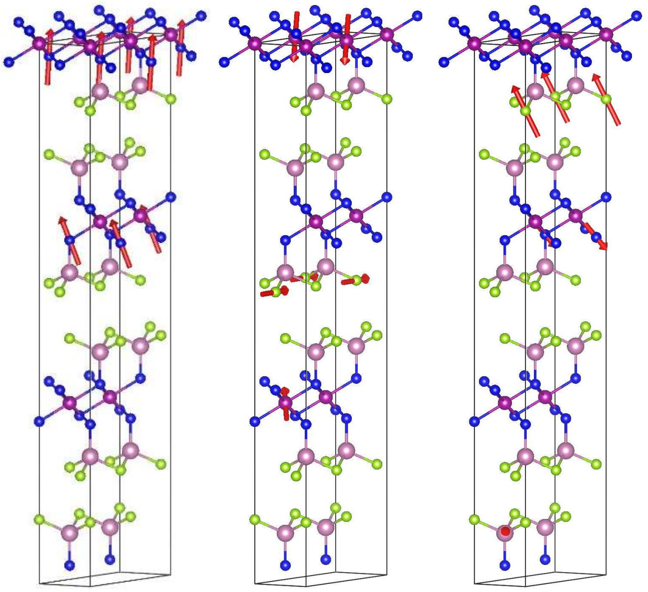

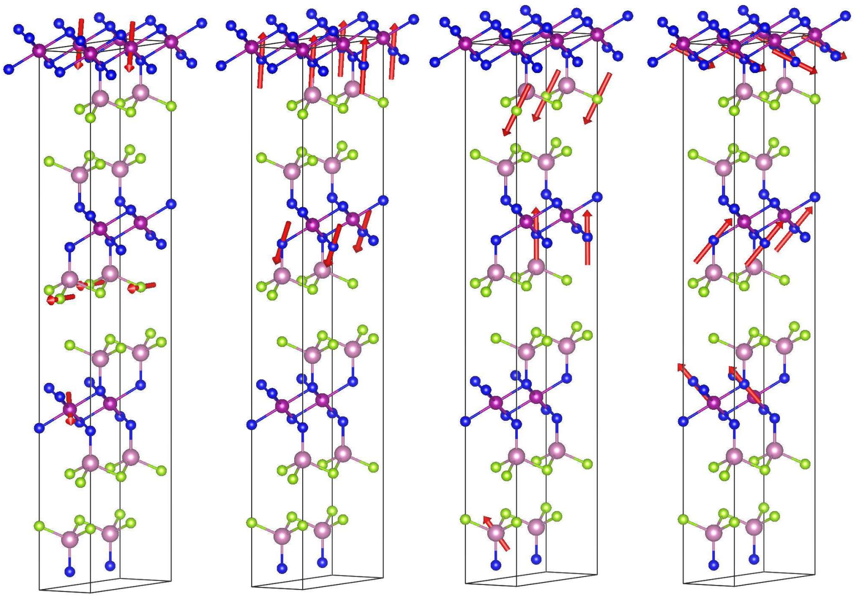

e)

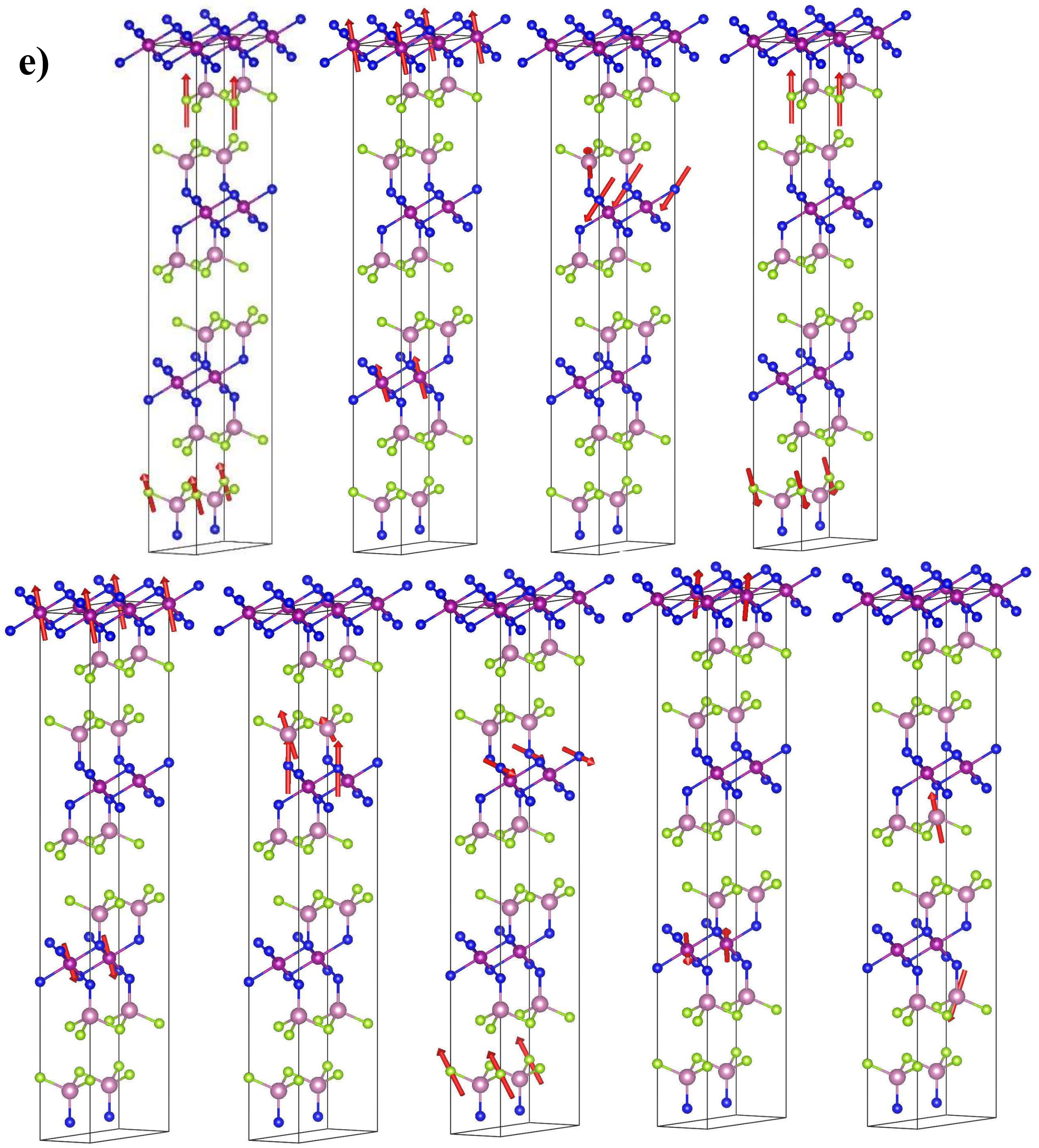

Figure S2. Phonon modes of $MnIn_2Se_4$ calculated by DFT. The modes are grouped into five frequency ranges: **a)** 6.5 cm^{-1}, **b)** 21 cm^{-1}, **c)** 62 cm^{-1}, **d)** 86 cm^{-1}, and **e)** 165 cm^{-1}.

Table S1. EDS elemental quantification obtained extracted from the flake shown in **Fig. 4**.

Element	Atomic Fraction (%)
Mn	13.1 ± 2.1
Se	59.0 ± 11.4
In	27.9 ± 5.1

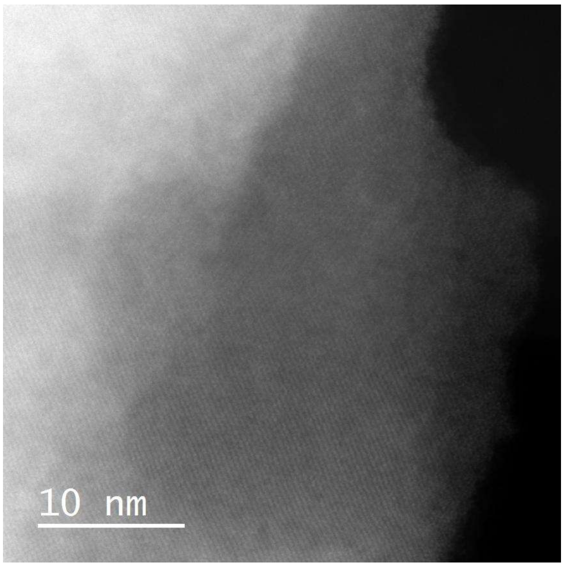

Figure S3. STEM-HAADF image of $MnIn_2Se_4$ flake shown in **Fig. 4**.

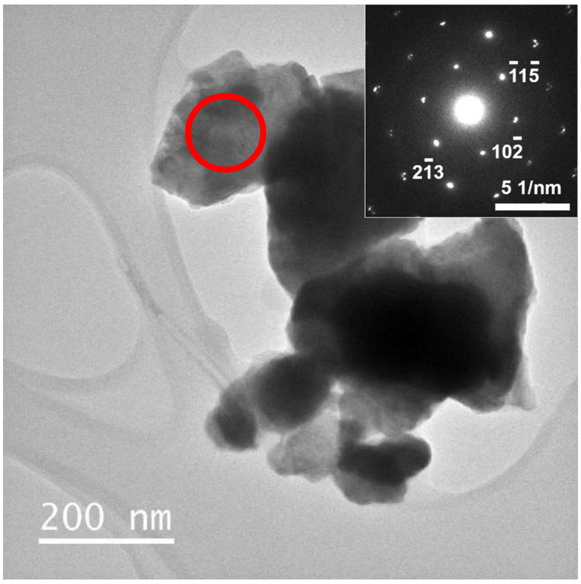

Figure S4. Low-magnification TEM image of $MnIn_2Se_4$ flakes. The red circle highlights the area used to perform the SAED analysis. The inset displays the SAED pattern which has been successfully indexed as belonging to the $MnIn_2Se_4$ *R3m* crystal structure seen along the [2 7 1] (=[-1 4 -3 1]) zone axis.

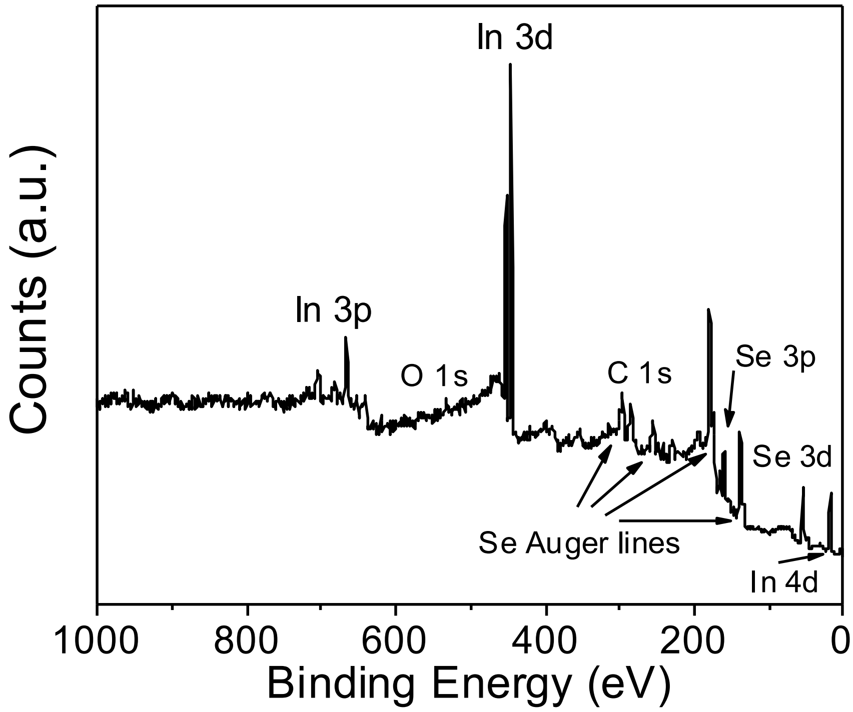

Figure S5. Wide-survey XPS spectrum of $MnIn_2Se_4$. Peaks marked with an arrow have arisen due to the Se LMM Auger transition.

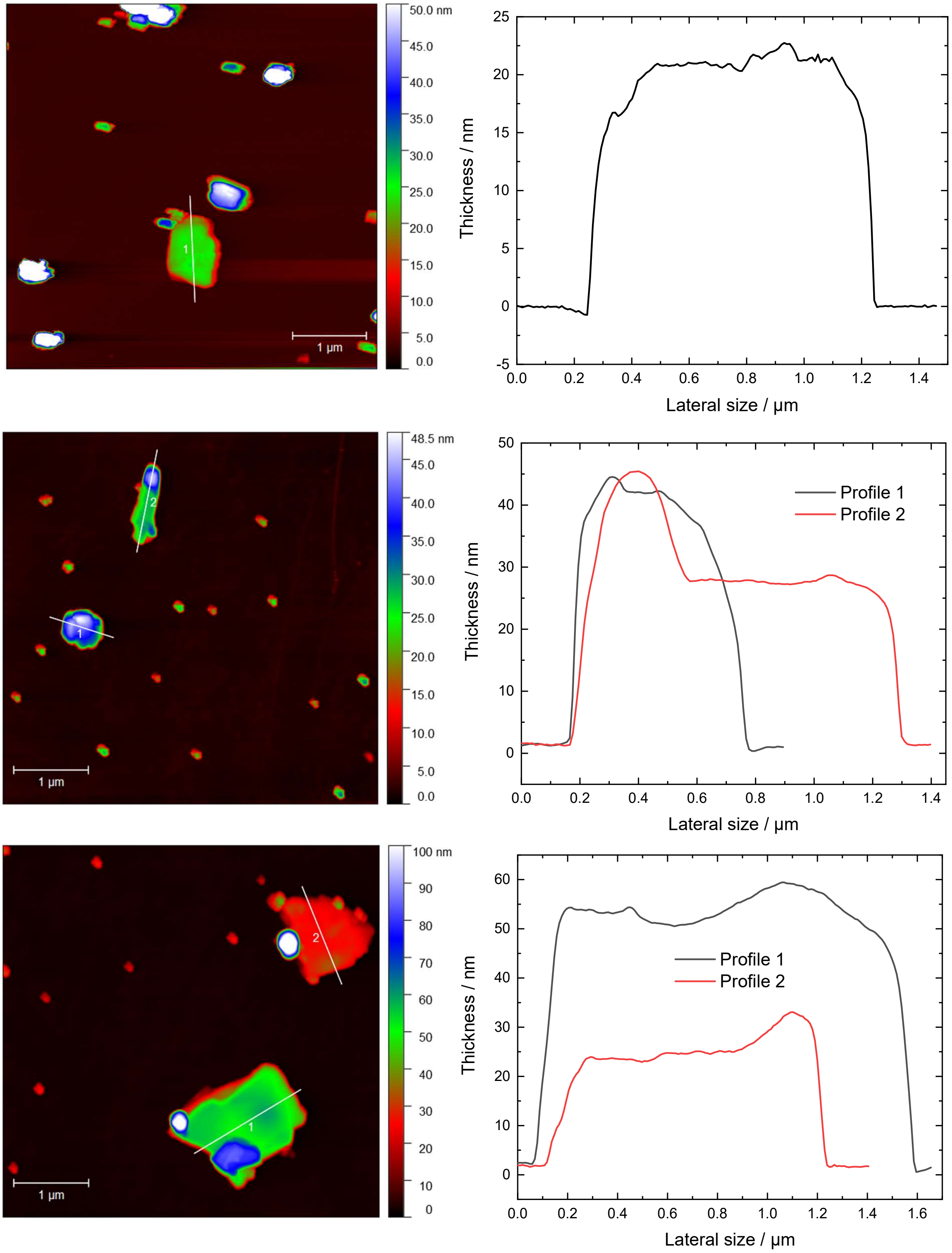

Figure S6. AFM images and the corresponding height profiles of various exfoliated $MnIn_2Se_4$ flakes.

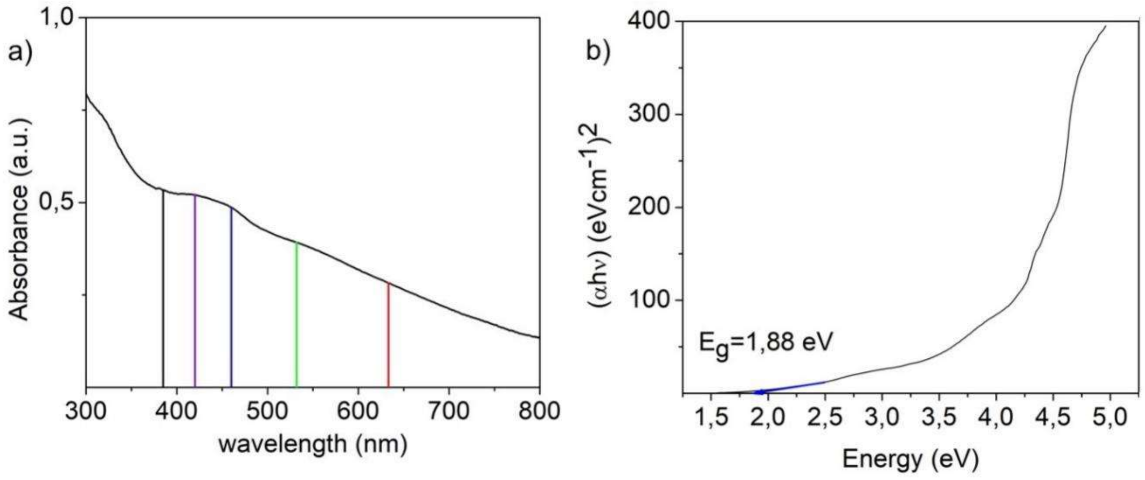

Figure S7. a) UV-Vis absorbance spectrum of the exfoliated $MnIn_2Se_4$ DMF suspension. The wavelengths of the LED light sources employed for the PEC study are marked in the graph. **b)** Tauc plot used to extract the optical band gap of $MnIn_2Se_4$ nanosheets.

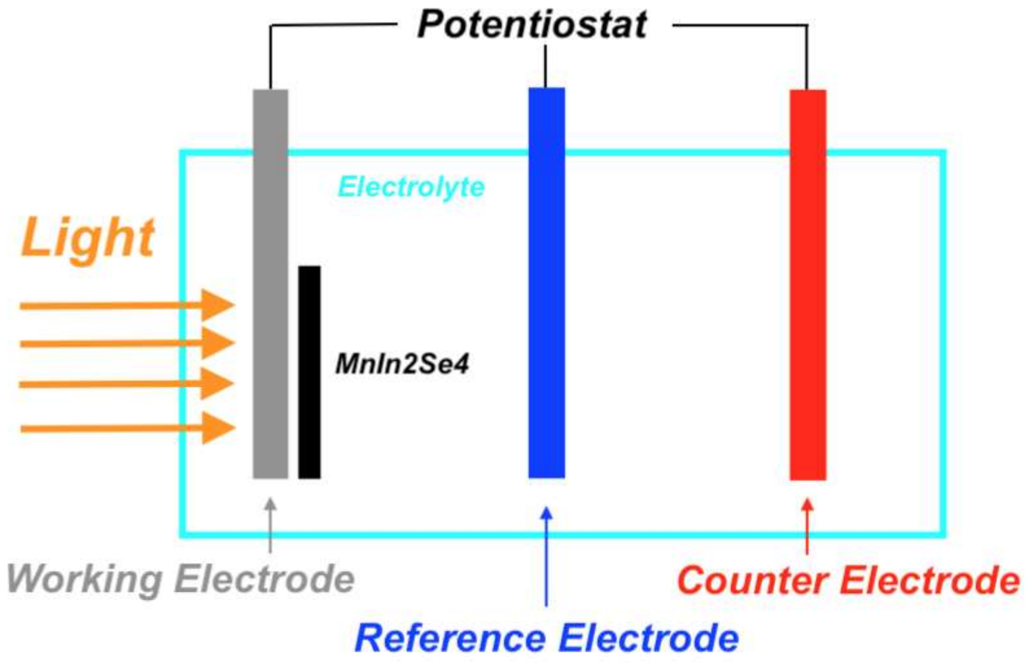

Figure S8. Schematic of the experimental setup for the PEC detector.

Table S2. Noise equivalent power (NEP) values for the $MnIn_2Se_4$ photodetector at different wavelengths and power levels, illustrating its noise characteristics.

Power Density (mW)	NEP ($W\ Hz^{-1/2}$)				
	UV Light 385 nm	Purple Light 420 nm	Blue Light 460 nm	Green Light 532 nm	Red Light 633 nm
1000	2.59×10^{-8}	1.17×10^{-10}	2.24×10^{-10}	3.21×10^{-9}	5.34×10^{-9}
800	2.90×10^{-8}	9.69×10^{-11}	1.97×10^{-10}	2.95×10^{-9}	4.88×10^{-9}
500	2.97×10^{-8}	7.60×10^{-11}	1.63×10^{-10}	2.46×10^{-9}	3.73×10^{-9}
300	2.82×10^{-8}	5.96×10^{-11}	1.36×10^{-10}	2.04×10^{-9}	3.13×10^{-9}
200	2.70×10^{-8}	4.99×10^{-11}	1.18×10^{-10}	1.73×10^{-9}	2.74×10^{-9}
100	2.63×10^{-8}	3.96×10^{-11}	9.74×10^{-11}	1.37×10^{-9}	2.17×10^{-9}
50	2.57×10^{-8}	3.07×10^{-11}	7.87×10^{-11}	9.90×10^{-10}	1.59×10^{-9}

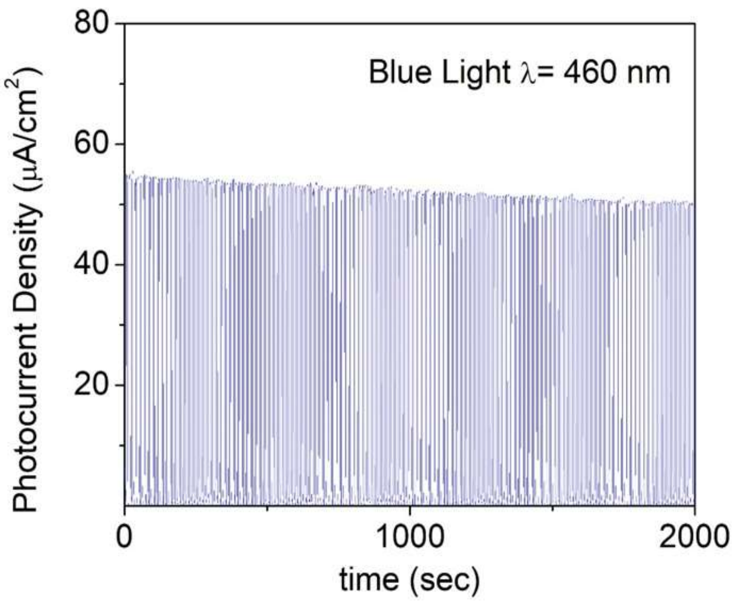

Figure S9. Long-term cycle stability test of $MnIn_2Se_4$-based photodetector in 1 M KOH ethanol (25% v/v) water solution at 0.5 V vs SCE under blue light, with irradiance power of 800 mW.

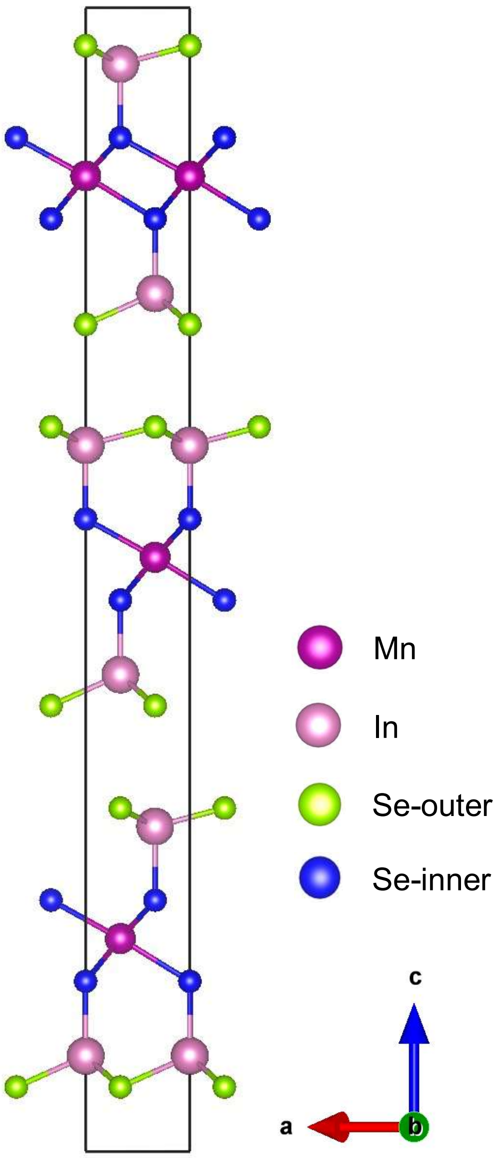

Figure S10. The $MnIn_2Se_4$ structure used in our DFT calculations. The manganese and indium atoms are indicated in purple and pink balls, respectively. Selenium atoms are separated into outer and inner atoms; the former indicate the atoms having only chemical bond with indium atoms, the latter are instead the atoms having chemical bond with both manganese and indium atoms. The outer and inner selenium atoms are indicated by green and blue balls, respectively.
